# Antibody Arrays Identify Potential Diagnostic Markers of Hepatocellular Carcinoma

**DOI:** 10.4137/bmi.s595

**Published:** 2008-01-21

**Authors:** Hongbo Sun, Mei-Sze Chua, Dorothy Yang, Anya Tsalenko, Brian J. Peter, Samuel So

**Affiliations:** 1 Asian Liver Center, Department of Surgery, Stanford University School of Medicine, Stanford, CA 94305; 2 Agilent Technologies, 5301 Stevens Creek Blvd., Santa Clara, CA 95051

**Keywords:** hepatocellular carcinoma, diagnosis, biomarkers, beta-catenin, protein arrays

## Abstract

Hepatocellular carcinoma (HCC) is the third leading cause of cancer deaths worldwide. Effective treatment of HCC patients is hampered by the lack of sensitive and specific diagnostic markers of HCC. Alpha-fetoprotein (AFP), the currently used HCC marker, misses 30%–50% of HCC patients, who therefore remain undiagnosed and untreated. In order to identify novel diagnostic markers that can be used individually or in combination with AFP, we used an antibody array platform to detect the levels of candidate proteins in the plasma of HCC patients (n = 48) and patients with chronic hepatitis B or C viral infections (n = 19) (both of which are the major risk factors of HCC). We identified 7 proteins that significantly differentiate HCC patients from hepatitis patients (p < 0.05) (AFP, CTNNB, CSF1, SELL, IGFBP6, IL6R, and VCAM1). Importantly, we also identified 8 proteins that significantly differentiate HCC patients with ‘normal’ levels of AFP (< 20 ng/ml) from hepatitis patients (p < 0.05) (IL1RN, IFNG, CDKN1A, RETN, CXCL14, CTNNB, FGF2, and SELL). These markers are potentially important complementary markers to AFP. Using an independent immunoassay method in an independent group of 23 HCC patients and 22 hepatitis patients, we validated that plasma levels of CTNNB were significantly higher in the HCC group (p = 0.020). In conclusion, we used an antibody array platform to identify potential circulating diagnostic markers of HCC, some of which may be valuable when used in combination with AFP. The clinical utility of these newly identified HCC diagnostic markers needs to be systematically evaluated.

## Introduction

Biomarker discovery is a burgeoning area of cancer research which encompasses the search for new diagnostic, prognostic, and treatment response markers of cancers. The ability to find new diagnostic markers of early stage cancers in particular holds great promise for more efficacious management of cancers that are hard-to-treat and that have poor patient survival, such as hepatocellular carcinoma (HCC). HCC is the third leading cause of cancer deaths worldwide (1). Its high mortality is in part due to limitations in early diagnosis of the malignancy. Patients often do not have overt symptoms until the cancer has progressed into advanced stages, limiting treatment options and resulting in poor prognosis. Patients with advanced HCC have substantially lower 5-year survival than those with early-stage disease (2). Given the markedly better prognosis with localized than distant disease, screening for early stage disease may offer the opportunity to improve the clinical management of HCC.

The major risk factors of HCC are chronic infections with hepatitis B or hepatitis C virus (HBV or HCV respectively). Chronic hepatitis can progress into cirrhosis (severe scarring or fibrosis of the liver), which increases the risk of developing HCC (3). Patients with chronic hepatitis and/or cirrhosis therefore form a high risk population which would benefit from regular screening for HCC by serial measurement of serum alpha-fetoprotein (AFP) levels and hepatic ultrasound (4, 5). AFP is a fetal glycoprotein produced by the yolk sac and fetal liver. Its serum levels usually decrease immediately after birth and then increase only in certain pathologic conditions, including HCC (4, 6). Elevated serum AFP (e.g. levels > 20 ng/ml) is not a specific marker for HCC, since it is detected in a wide variety of non-hepatic malignancies (7–9) and benign conditions, including acute and chronic hepatitis (10–15). In chronic hepatitis carriers, the specificity of AFP for HCC ranges from 80% to above 90%, but its positive predictive value is well below 10% (5, 16, 17). Furthermore, the sensitivity of AFP as an HCC marker is also limited, since 30%–50% of HCC cases do not present with elevated serum AFP (2). It is unclear whether the sensitivity or specificity of AFP varies among HBV-positive, HCV-positive, and non-viral HCC (18–22); however, it is apparent that serum AFP fails to detect a substantial proportion of HCC of any etiology.

The sensitivity of ultrasound imaging for detection of HCC tumor nodules is also limited, ranging from 35% to 81% and varying by operator and hospital (23). Moreover, because lesions identified with ultrasound are frequently re-examined by computed tomography (CT) scan, the cost of radiographic tests for HCC is high (3). Even with helical CT, approximately 30% of tumors smaller than 2 cm may escape detection (23), again limiting the utility of radiographic screening for early HCC. Therefore, screening methods with improved sensitivity and specificity are critical to the reliable diagnosis of early stage HCC, which is fundamental to improving patient survival.

In this study, we used an antibody array that measured 75 independent serum proteins to identify potential circulating diagnostic markers of HCC. Because chronic hepatitis is an inflammatory condition which predisposes to HCC, we used arrays consisting largely of antibodies against inflammatory proteins to probe for those which may be useful for diagnosing the transition from hepatitis to HCC. Proteins found to significantly differentiate HCC from hepatitis may be valuable as early diagnostic markers for the routine screening of patients with chronic hepatitis.

## Patients and Methods

### Patient samples

A total of 112 patients were recruited at Stanford University Hospital between April 2003 and August 2005. Of these, 67 patients were used to generate protein expression profiles using the antibody arrays (19 patients with chronic hepatitis B or C viral infections; 48 HCC patients) (Protein Array Group). The remaining 45 patients (22 patients with chronic hepatitis B or C viral infections; 23 HCC patients) were used as an independent sample set for validation purposes (Validation Group). The patient characteristics of each group are shown in Table 1. Prior to blood draws, all patients signed informed consent approved by the Stanford Institutional Review Board for the use of human subjects in medical research. The age range of the sample population is 25–81 years. HCC patients were defined as those with a pathological diagnosis of HCC after surgery or biopsy or with an evidence of tumor on CT or angiography. Patients with chronic HBV or HCV were confirmed to have no history of HCC and no radiographic evidence of tumor; they were followed up for at least 6 months to exclude individuals with asymptomatic HCC. All blood samples were collected in purple top Vacutainer tubes using EDTA as an anticoagulant, and plasma separated after 15 min centrifugation at 1000x g. Plasma samples were stored at −70°C before assay.

### Antibody array fabrication

The antibody arrays consist of pairs of capture and detection antibodies against 75 unique proteins, including inflammatory proteins, tumor-specific proteins, and AFP (Table 2; see also supplemental data). The antibody pairs were purchased from R&D Systems, Inc. (Minneapolis, MN). Antibodies provided in trehalose were dialyzed into a trehalose-free, phosphate-buffered saline (PBS) solution using centrifugal filtration tubes with molecular weight cut-off of 5000 Daltons. An Agilent inkjet-based deposition system (24) was used to spot the capture antibody solutions in quadruplicates on 1 × 3 inch chemically modified glass substrates at two concentrations (250 μg/mL and 500 μg/mL), with 8 antibody arrays per slide. The resulting microarrays were sealed in a slide box and stored under nitrogen at room temperature. Further details of the antibody array manufacturing process, and characteristics of the arrays can be found in the Supplemental Data.

### Antibody array processing

All binding and washing steps were perfomed at room temperature. The antibody arrays were placed in 12% non-fat milk for 10 min to remove unbound antibodies and to minimize non-specific adsorption. Excess milk was removed by washing twice in PBS with 0.05% Tween-20 (PBST), once in PBS (2 min each) and finally in distilled water for 1 minute. The slides were then spun dry with a Beckman GPKR centrifuge at 1500 rpm for 2 min. Plasma samples were diluted three times with binding solution (0.4% triton X-100 and 1% casein block buffer (Pierce Biotechnology, Rockford, IL), and 40 μL of the diluted sample solution was applied to each chamber of the 8-pack Gasket Slide (Agilent Technologies, Santa Clara, CA). Sample sets were systematically distributed across arrays, so that each array contained samples from each of the different patient groups. The antibody array was then placed over the gasket slide, and SureHyb Gasket Chamber (Agilent Technologies, Santa Clara, CA) was assembled to seal the slides. The slides were incubated overnight using gentle rotation at 4 rpm in Hybridization Incubator (Robbins Scientific, Model 400). After incubation, the arrays were rinsed in PBST, washed twice in PBST (10 min each), once in PBS (10 min), and once in distilled water for 1 minute before being spun dry. Next, biotinylated detection antibodies were diluted 1:90 with binding buffer (0.4% triton X-100 and 1% casein block buffer). The detection antibody mixture (40 μL) was used to bind to the antigen. After incubation for 2 hr, the arrays were washed and spun dry as described above. Then 40 μL (0.4 μg/mL) of fluorescent Cy3-labeled strepta-vidin (Sigma, St. Louis, MO) was added to detect the signal. After 30 min incubation, the slides were washed and spun dry as described above. Finally, the arrays were scanned in an Agilent G2565AA DNA Microarray Scanner (Agilent Technologies, Santa Clara, CA) at 100%, 10%, and 2% PMT individually using 5 μm resolution with only the green channel on. Each sample was measured on two separate slides to check for reproducibility. To assess background levels of nonspecific antibody binding to the arrays, eight “blank” arrays with buffer in the place of plasma were processed together with the other samples. Further measurements of background signal and antibody cross reactivity are presented in the Supplemental Data.

## Data Extraction

The location of each antibody spot on the array was outlined using Agilent Feature Extraction software version A.7.5.1 (Agilent Technologies, Santa Clara, CA). The signals for each spot were obtained by subtracting the median of pixel intensities from the local area around each spot from the average pixel intensity within each spot. The array elements for which the fluorescent intensity of each spot was less than 3 times the standard deviation of the blank controls were excluded. Each antibody is represented by four spots on each array; if less than 2 spots were located by the Feature Extraction Software, the array element was excluded from further analysis. The median value was taken from the located spots for each antibody, and the median values from replicate arrays were averaged. For each antibody, the median of the signals from the blank arrays (representing background from antibody cross-reaction) was subtracted from the average intensity of each plasma sample. Details of the measurement and calculation of background signals can be found in the Supplemental Data. Data were log transformed before further analysis.

## Bioinformatics Analyses

We initially identified proteins that have statistically significant differential expression between two or more groups of samples using ANOVA and Student’s t-test (25). Differences were considered statistically significant if the P value was less than 0.05. In addition to evaluating differential expression for each protein independently, we used leave one out cross validation analysis to identify sets of proteins that together can predict disease status of the patient (25).

## Beta-Catenin Validation by Immunoassay

Beta-catenin (CTNNB) plasma concentrations were determined using enzyme immunometric assay kits from Assay Designs, Inc. (MI, U.S.A), according to the manufacturer’s instructions. The values were log transformed, and Student’s t-test was used to compare the variation between two groups. Difference was considered statistically significant if the P value was less than 0.05.

## Results

### Antibody arrays offer sensitive and reproducible protein detection

**Figure 1:**
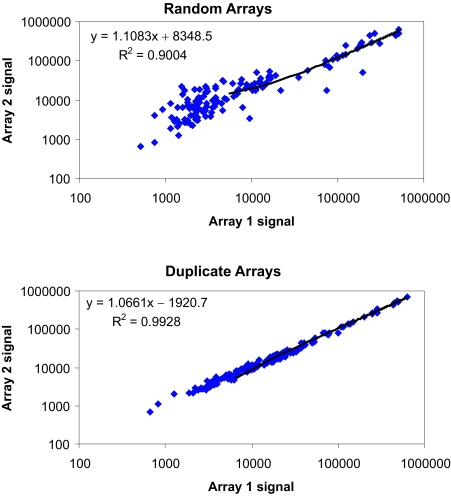
Duplicate arrays show greater correlation coefficients than a random pair of arrays Background-subtracted log-10-transformed fluorescence signals for each protein are shown with one sample shown on each axis. The tight distribution of data in the duplicate arrays indicates the platform reproducibility.

To test the sensitivity of our antibody arrays, serial dilutions of standard proteins were measured. The sensitivity of the array varied for different antibody pairs, but 40% of the antibody probes tested gave a response with 100 pg/ml of antigen or less (Table 3; see also Supplemental Data). For some proteins which are expressed at higher levels in plasma, even those antibody probes with lower sensitivities gave robust signals (e.g. IL1RN). Each sample was run on duplicate arrays on different slides. To check the reproducibility of our array data, we compared the correlation between duplicate arrays of the same patient with the correlation of two random arrays (Fig. 1). The average r-square value of 100 duplicates was 0.98, while the average r-square of 100 random pairs was 0.90. Additionally, 76% of the duplicate arrays had correlation coefficients greater than 0.99, and 97% had correlation coefficients greater than 0.95. Even duplicates in the lowest quartile of r-square values showed higher correlations on average than random pairs, indicating that platform variation was less than biological variation. Additional information describing the antibody array platform can be found in the Supplemental Data section.

### HCC can be significantly differentiated from chronic hepatitis

**Figure 2:**
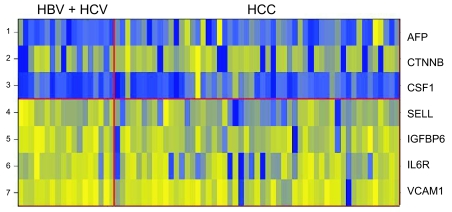
Heatmap for top scoring proteins that differentiate between HCC and hepatitis The yellow shade represents higher fluorescence signals for the protein and blue shade represents the lower signals for the proteins. AFP, CTNNB and CSF1 show up-regulation in HCC group, while SELL, IGFBP6, IL6R and VCAM1 show down-regulation in HCC group.

We used supervised learning methods to identify the proteins most highly variable between the two groups. Among the 75 unique probes, 7 showed statistically significant differential expression between HCC (n = 49) and hepatitis (HBV or HCV; n = 18) groups: AFP, beta-catenin (CTNNB), and colony stimulating factor-1 (CSF1) were up-regulated in HCC; L-Selectin (SELL), insulin growth factor binding protein-6 (IGFBP6), interleukin 6 receptor (IL6R), and vascular cell adhesion molecule 1 (VCAM1) were very slightly down-regulated in HCC (p < 0.05) (Table 4, Fig. 2).

Although these proteins were expressed differentially between the HCC versus hepatitis groups, each by itself was not a perfect classifier for the prediction of HCC, and the magnitude of the differences was often low. However, the combined pattern of four proteins, AFP, SELL, IGFBP6, and IL6R, can create a high quality predictor. The robustness of the classifier to separate the two groups was investigated using a weighted-voting algorithm and evaluated by cross validation testing (25). A positive predictive value of 78% (55 out of 71 correct classifications) for the study population was obtained.

### HCCs with AFP <20 ng/ml can be significantly differentiated from chronic hepatitis

**Figure 3:**
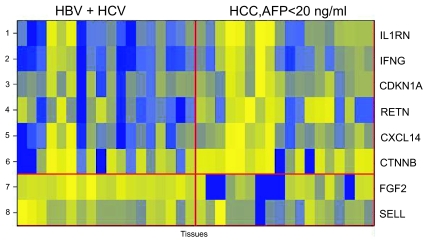
Heatmap for top scoring proteins that differentiate between HCC with AFP <20 ng/ml and hepatitis The yellow shade represents higher fluorescence signals for the protein and blue shade represents the lower signals for the proteins. IL1RN, IFNG, CDKN1A, RETN, CXCL14 and CTNNB show up-regulation in HCC group with normal AFP level (<20 ng/ml). FGF2 and SELL show down-regulation in HCC group with normal AFP level (<20 ng/ml).

Among the 75 unique probes, 8 showed statistically significant differential expression between specimens from HCC patients with ‘normal’ serum AFP <20 ng/ml (n = 16) and specimens from hepatitis patients (with HBV or HCV infections) (n = 18): interleukin-1 receptor antagonist (IL1RN), interferon-gamma (IFNG), cyclin-dependent kinase inhibitor 1A (CDKN1A), resistin (RETN), chemokine (C-X-C motif) ligand 14 (CXCL14), and CTNNB were up-regulated in HCC with low AFP, whereas fibrolast growth factor-basic (FGF2), and SELL were down-regulated in HCC with low AFP (p < 0.05) (Table 5, Fig. 3).

A classifier made from two proteins, CDKN1A and tumor necrosis factor-beta (TNFB), was created using the forward search method. A positive predictive value of 80% (27 correct classifications out of 34) was achieved by cross validation. TNFB by itself was not a significant distinguisher between these two groups of patients (p = 0.073).

### Independent Immunoassay Testing of a Potential Biomarker

**Figure 4:**
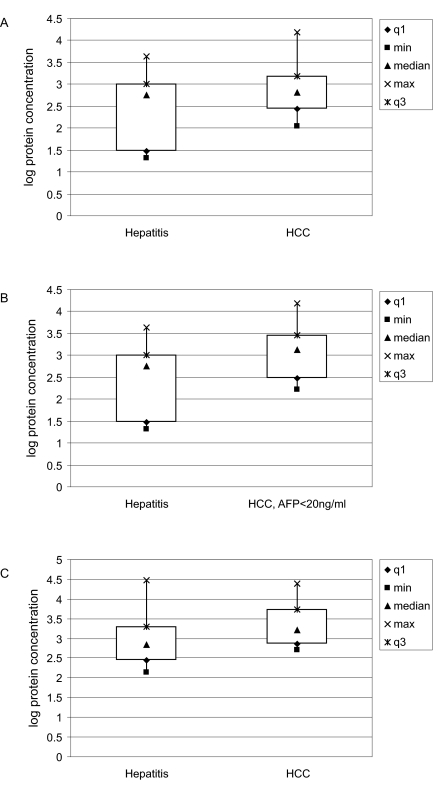
Box plots showing the distribution of measurements of CTNNB serum levels by an independent immunoassay The median values are indicated by triangles, the minimum and maximum values are squares and crosses, respectively, and the box defines the boundaries of the first and third quartiles of data. Sample groups are on the x axis and the logarithm of the measured protein concentration is on the y axis. (**A**). Samples (42 HCC and 15 hepatitis patients) were randomly selected from the Protein Array Group, and CTNNB levels measured to be statistically significant between the 2 groups (p = 0.025). (**B**). Among these samples, CTNNB levels were also statistically significant between HCC patients with AFP <20 ng/ml (n = 16) and hepatitis patients (n = 15) (p = 0.036). (**C**). Significant differential levels of CTNNB were confirmed in an independent Validation Group consisting of 23 HCC and 22 hepatitis patients.

To begin to test the robustness of the biomarkers we discovered on the antibody array platform, we used an independent immunoassay for CTNNB, which showed the greatest fold-change between HCC and hepatitis (Tables 4 and 5). We measured the levels of CTNNB in an ELISA which used a different antibody pair than those on the array (further examples of ELISA/array comparisons are shown in Supplemental Data). Specimens from HCC patients (n = 42) and from hepatitis patients (n = 15) were randomly selected from the Protein Array Group, and their CTNNB levels were measured using commercially available ELISA kits from Assay Designs, Inc. Although correlation of immunoassay results with that of antibody array results was poor (R^2^ = 0.375), the expression level of CTNNB in HCC (median = 651 pg/ml) was significantly higher than that in the hepatitis group (median = 575 pg/ml) (p = 0.025) (Fig. 4A). Additionally, when comparing 16 HCC patients with low AFP (AFP < 20 ng/ml) to 15 patients with chronic hepatitis, CTNNB remains significantly elevated in the group of HCC patients with AFP < 20 ng/ml (median = 1307 pg/ml) (p = 0.036) (Fig. 4B). We found similar results in the Validation Group: CTNNB was significantly more abundant in 23 HCC patients (median = 1652 pg/ml) compared to 22 chronic hepatitis patients (median = 680 pg/ml) (p = 0.020) (Fig. 4C). However, because of small sample numbers, the comparison of CTNNB levels between HCC patients with AFP < 20 ng/ml and hepatitis patients (n = 6) only approaches significance (p = 0.07) (data not shown).

Although the magnitudes of the ELISA signals only show a weak correlation to the array signals, the results are consistent with our antibody array data, implying that beta-catenin may be a potential diagnostic marker for HCC, including HCC with undiagnostic levels of AFP.

## Discussion

Using an antibody array technology, we identified circulating markers that distinguished HCC patients from patients with chronic hepatitis. We used an independent immunoassay method to confirm that one of the markers, CTNNB, shows differential expression when measured with antibodies from a different supplier. The protein markers that we identified have little or no prior association with HCC, and are thus novel candidates as diagnostic markers for HCC. Importantly, some of these markers (e.g. CTNNB) could potentially be useful for detecting HCC in patients with lower than diagnostic levels of AFP.

The antibody array that we used includes antibodies against proteins involved in inflammation (such as cytokines, chemokines, interleukins, interferons); proteins involved in cell adhesion and the extracellular matrix; proteins involved in apoptosis; growth factors and their receptors and binding proteins, and AFP. Since chronic hepatitis infection is the major risk factor for the development of HCC, inflammatory proteins might be expected to be elevated in the early transition from hepatitis to HCC, and their over-abundance in HCC might be useful diagnostic indicators for this hard-to-detect malignancy. Whereas some of these inflammatory proteins might indeed have some causative roles in the development of HCC, others might be indicators of the host response to the infection or tumor. Clearly, the multivariate analysis enabled by antibody arrays offers better power than individual measurements for classifying the samples, irrespective of whether the mechanism of the individual proteins in HCC is known.

Our identification of AFP as a marker that is over-expressed in HCC confirms the robustness of our technology and bioinformatics analyses. Among 8 potential markers of HCC, only AFP and CTNNB are more than twofold more abundant in HCC compared to hepatitis. While the magnitude of the expression differences was low for the other potential markers, the low p-values indicate that the small changes may be significant when detected on a platform with low noise. Although SELL most significantly distinguishes HCC patients from hepatitis patients, it is less abundant in HCC. Intuitively, proteins that are under-expressed in HCC compared to hepatitis appear to have less clinical value than those that are over-expressed in HCC. However, this does not negate their potential clinical utility as diagnostic and therapeutic markers, given that other established diagnostic or prognostic tests are based on low marker levels (e.g. CD4+ T-cell count, white blood cell count, or thyroid hormone concentration). It is also conceivable that a panel of markers, some of which are up-regulated and some of which are down-regulated in HCC, might have greater diagnostic value than a single marker alone.

CTNNB and SELL appear to have the potential to distinguish not only HCC patients from hepatitis patients, but also HCC patients with AFP < 20 ng/ml from hepatitis patients. Proteins that uniquely distinguish HCC patients with low AFP from patients with hepatitis include IL1RN, IFNG, CDKN1A, RETN, CXCL14, and FGF2. These protein markers have potentially important clinical impact in the detection of HCC patients that are undiagnosed due to their low levels of AFP.

CTNNB stands out as a promising marker for HCC, as well as for HCC with low AFP, since it shows at least 3 fold abundance over patients with chronic hepatitis. Furthermore, its significant over-abundance in both groups of HCC patients was validated by independent immunoassay method. CTNNB is an adherent junction protein that helps to mediate adhesion between cells, and to maintain epithelial layers such as those lining organ surfaces (http://genome-www5.stanford.edu/cgi-bin/source/sourceSearch). Tumor cell metastasis, which involves the disruption and reestablishment of epithelial cell-cell contacts, may be regulated by the disassembly and assembly of adherent junctions. Activating mutations in CTNNB have oncogenic activity and have been found in various types of tumors, such as colon, ovarian, prostate, and liver. CTNNB also regulates signal transduction through the Wnt pathway, which is known to be dysregulated in various types of cancers, including HCC and melanoma (26, 27).

The expression of CTNNB in HCC has been detected immunohistochemically by several groups. Mutated nuclear CTNNB over-expression has been associated with increased cell proliferation (28) and poorer survival (29). Non-nuclear types of CTNNB over-expression (in the cytoplasm and/or cytoplasmic membrane) may also have pathologic and prognostic significance, being associated with tumor size >5 cm, poorer cellular differentiation, and shorter disease-free survival (30). However, there has been no report of serum detection of CTNNB in HCC, nor of its associations with clinicopathological features of HCC. Given the important biological implications of CTNNB in HCC, the ability to detect it in peripheral blood of HCC patients would offer a valuable, non-invasive way of HCC diagnosis and prognostication. Our data further suggests that CTNNB could be a useful complementary marker to AFP in the diagnosis of HCC.

Our current study is the first of many steps in the identification of novel diagnostic markers for HCC (31). Pending validation of these results by immunoassays in an independent set of samples, each potential marker needs to undergo further evaluation steps as outlined below (32): 1). a “preliminary performance” study comparing the performance of the marker between subjects with and without symptomatic cancer; 2). a “retrospective performance” study evaluating the performance of the marker in stored, pre-disease specimens from asymptomatic subjects who went on to develop cancer, compared to those who did not; 3). a “prospective performance” study evaluating the performance of the marker in a follow-up cohort of asymptomatic subjects, comparing those who are later diagnosed with cancer to those who are not; and 4). a “cancer screening” study, ideally in the form of a randomized clinical trial, evaluating the clinical utility, benefits, and harms of the marker as the basis for early intervention (31, 32).

In conclusion, we used arrays of antibodies against inflammatory proteins to identify novel potential circulating markers of HCC. Importantly, several of these markers are elevated in HCC patients with non-elevated AFP, and therefore, may be used clinically to complement the current AFP diagnostic test for more accurate detection of HCC. The clinical value of these markers, and especially of beta-catenin, warrants further large scale validation.

## Supplementary Information

### Supplemental data

#### I. Details of antibody array manufacturing

**Figure S1:**
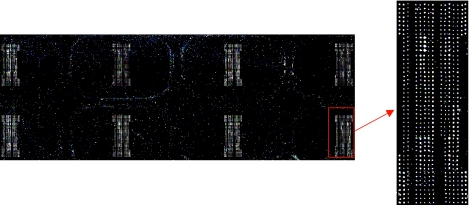
Agilent 8-pack antibody array A scanned fluorescent image of 8 protein arrays on one slide is shown; one array is enlarged to show detail. On a typical array, background signals in between the antibody features ranged from 30–60 fluorescent units, while the signals on the antibody features ranged from 200–400000.

The Agilent deposition system is comprised of an engineered print head that can hold sixty different solutions and dispense ~40 picoliters per spot. The precision and accuracy of liquid dispensing is computer controlled. The printing was performed on 12 × 12 inch glass wafers which were then diced into 1 × 3 inch slides. The system prints multiple wafers with the same load of antibody solutions. The content can be composed of antibodies, full-length proteins, protein fragments; further details of the system can be found in reference 24 of the paper. In our study, we printed antibodies on an Agilent proprietary chemically modified surface. The antibodies adsorbed to the surface via non-covalent interactions. We designed the arrays such that one 1 × 3 inch slides holds 8 identical arrays for 8 samples or controls (Fig. S1). Each antibody was printed in four replicates at two different concentrations per array for statistical calculations. Samples for the array can range from discrete proteins to complex biological matrices such as serum or cell lysates. The array was processed under the protocol described in the paper using Agilent Sure Hyb chambers and 8-pack gaskets.

#### II. Background signal and antibody cross-reactivity

**Figure S2:**
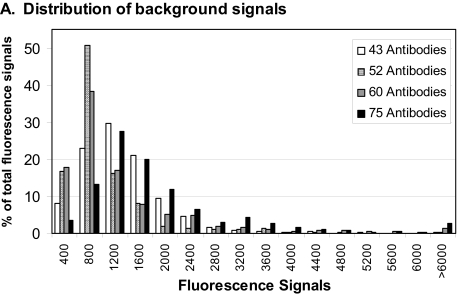

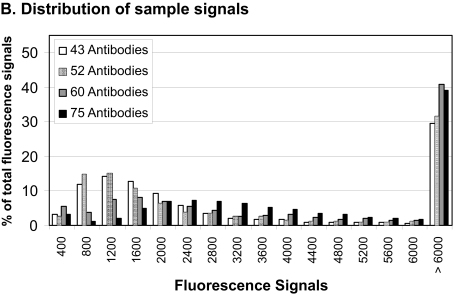
Distributions of signals from samples versus background signals with different levels of multiplexing **A** Histograms of the signal distributions from buffer blanks using four different detection antibody mixtures are shown. As the number of antibodies in the detection mixture increased, the background fluorescence in the array features increased, but the peak of the distribution remained >1500. **B.** Histograms of signals from all sample arrays for the four different detection antibody mixtures are shown with the same scale as A. For data analysis, we calculated significant background-subtracted signals using three standard deviations of the blank signal for each antibody. However, it is clear from these plots that very few antibodies had fluorescence signals >5000 in the absence of samples, while many high signals were measured when actual samples were applied.

As the number of antibodies increases, the potential for antibody or antigen cross-reactivity also rises. We addressed this by doing extensive quality control experiments for each new antigen and antibody pair that was added to the arrays; antigen/antibody sets which led to a significant increase in signal in the absence of antigen were left off of the array and out of the detection antibody mixture. Furthermore, for each experiment we measured the feature background signal with a “blank” array which had only buffer and detection antibodies applied; if the detection antibody mixture showed significant fluorescent signal in the absence of sample, it was likely that some detection antibodies in the mixtures were binding directly to the capture antibody spots. The substrate background signals from proteins binding where no capture antibody was printed were consistently very low (30–60) and were not included in further calculations. The feature background signals and actual sample signals were different for each antibody pair, and thus the background-subtracted signal was calculated independently for each antibody pair (see methods). In general we found that the feature background signals increased as the number of antibodies in the detection mixture increased (Fig. S2 A), but we found the feature background signals to be manageable with careful selection of antibody reagents. Also, we performed an independent experiment using the same sample with mixtures of either 40 or 60 detection antibodies, and measured the increase in signal in the buffer blank and sample arrays. We found that the extra 20 antibodies into the mixture only increased the median background signal by 300, while the significant signals from plasma samples were usually in the range of 5000–200000 (Fig. S2 B). Finally, because the actual levels of antigen could be very low in many samples, a failure to detect signal above background does not mean that the antibody pair is not working.

We further quantitated the level of cross-reactivity by measuring specific and nonspecific signals during antigen titrations (data not shown). While we usually saw a large, linear increase in fluorescence at the array feature for the specific antibody (Fig. S4), there was rarely any significant increase in signal in the nonspecific features.

#### III. Protein array sensitivity—Antigen titration curves and comparison to ELISA results

**Figure S3:**
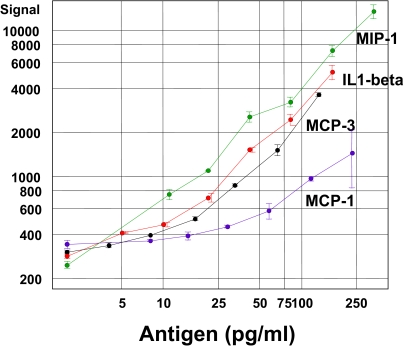
Examples of Antigen titrations Purified antigen proteins in buffer were applied to protein arrays, and the relative fluorescent signal was measured. Many proteins showed approximate limits of detection (LOD) <100 pg/ml.

**Figure S4:**
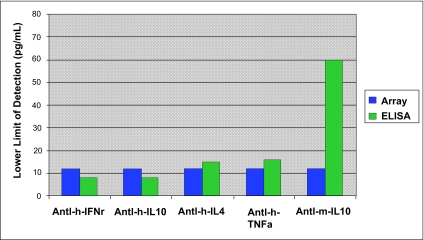
Limit of detection for ELISA versus protein array LODs were measured with purified antigen titrations on the two platforms, using ELISA kits constructed with the same antibody pairs used on the protein array.

**Figure S5:**
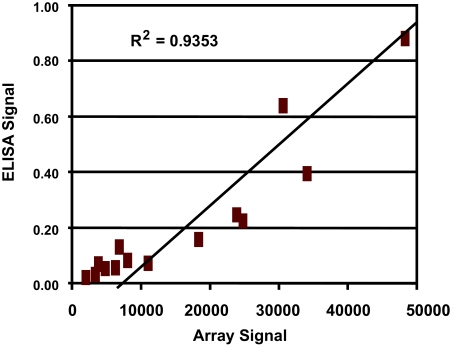
ELISA versus protein array results for the protein Leptin Relative fluorescent signals from ELISA and the protein array are plotted. The ELISA kit used the same antibody pairs as the protein array.

Once the array was printed, each antibody was tested for its response in terms of fluorescent signal as a function of specific antigen concentration in order to determine the sensitivity and dynamic range of array (Fig. S3). More limit of detection (LOD) data can be found in Table 2. The array performance was evaluated against ELISA using the same capture antibody and biotinylated detection antibody pair (Fig. S4 and S5). In general, the array provided similar LOD as ELISA if not better, but with much broader dynamic range.

## Figures and Tables

**Table 1:** Patient characteristics in Protein array group and validation group.

	Protein array group				Validation group			
	HCC		HBV and/or HCV carrier		HCC		HBV and/or HCV carrier	
		
	N	(%)	N	(%)	N	(%)	N	(%)
**Age group (years)**								
<50	9	(18.8)	5	(26.3)	3	(13.0)	11	(50.0)
≥50	39	(81.2)	14	(73.7)	20	(87.0)	11	(50.0)
**Gender**								
Male	39	(81.3)	14	(73.7)	19	(82.6)	13	(59.1)
Female	9	(18.8)	5	(26.3)	4	(17.4)	9	(40.9)
**Race**								
Asian/Pacific Islander	34	(70.8)	11	(57.9)	20	(87.0)	16	(72.7)
White, non-Hispanic	11	(22.9)	8	(42.1)	3	(13.0)	6	(27.3)
Other/unknown	3	(6.3)	0	(0.0)	0		0	
**Hepatitis virus seropositivity (HCC cases only)**								
HBV-positive	26	(54.2)			17	(73.9)		
HCV-positive	10	(20.8)			4	(17.4)		
HBV- and HCV-positive	1	(2.1)			1	(4.3)		
None/unknown	11	(22.9)			1	(4.3)		
**Peak alpha-fetoprotein (ng/ml)**								
<20	18	(37.5)	17	(89.5)	5	(21.7)	8	(36.4)
20–199	16	(33.3)	2	(10.5)	8	(34.8)	0	(0.0)
≥200	13	(27.1)	0	(0.0)	10	(43.5)	0	(0.0)
Unknown	1	(2.1)	0	(0.0)	0	(0.0)	14	(63.6)
Median	39.7		4.9		69.4		5.27	

**Table 2:** List of antibodies included on arrays.

Gene symbol	Gene name	Biological process
ADIPOQ	Adiponectin	Metabolic and hormonal processes, phosphate transport
AFP	Alpha-fetoprotein	Immune response, transport
BCL2	B-cell CLL/lymphoma 2	Anti-apoptosis
BAX	BCL2-associated X protein	Apoptosis
CCL1	Chemokine (C-C motif) ligand 1	Calcium ion homeostasis, cell-cell signaling
CCL11	Chemokine (C-C motif) ligand 11	Calcium ion homeostasis, cell-cell signaling, cell adhesion
CCL13	Chemokine (C-C motif) ligand 13	Calcium ion homeostasis, cell-cell signaling
CCL18	Chemokine (C-C motif) ligand 18	Antimicrobial humoral response, cell-cell signaling
CCL2	Chemokine (C-C motif) ligand 2	G-protein signaling, anti-apoptosis, cell adhesion
CCL21	Chemokine (C-C motif) ligand 21	Cell-cell signaling, chemokine activity
CCL22	Chemokine (C-C motif) ligand 22	Antimicrobial humoral response, cell-cell signaling, chemokine response
CCL28	Chemokine (C-C motif) ligand 28	Chemokine activity, chemotaxis
CCL5	Chemokine (C-C motif) ligand 5	Cell adhesion and motility, cell-cell signaling, chemokine activity
CCL7	Chemokine (C-C motif) ligand 7	Antimicrobial humoral response, cell-cell signaling, chemokine response
CCL8	Chemokine (C-C motif) ligand 8	Cell-cell signaling, chemokine activity
CD36	Thrombospondin receptor	Transport, cell adhesion
CDKN1A	Cyclin-dependent kinase inhibitor 1A/p21	Apoptosis, cell cycle arrest
CSF1	Colony stimulating factor 1 (macrophage)	Cell differentiation and proliferation
CTNNB	Beta-catenin	Wnt receptor signaling pathway, cell adhesion
CX3CL1	Chemokine (C-X3-C motif) ligand 1	Cell adhesion, chemokine activity
CXCL1	Chemokine (C-X-C motif) ligand 1	G-protein coupled receptor protein signaling pathway
CXCL10	Chemokine (C-X-C motif) ligand 10	Cell-cell signaling, cell motility, chemokine activity
CXCL11	Chemokine (C-X-C motif) ligand 11	Cell-cell signaling, chemokine activity
CXCL12	Chemokine (C-X-C motif) ligand 12	G-protein coupled receptor protein signaling pathway, cell adhesion, chemokine activity
CXCL14	Chemokine (C-X-C motif) ligand 14	Cell-cell signaling, chemokine activity
CXCL9	Chemokine (C-X-C motif) ligand 9	G-protein coupled receptor protein signaling pathway
EGF	Epidermal growth factor	DNA replication, activation of MAPK
EGFR	Epidermal growth factor receptor	ATP binding, cell cycle, cell proliferation
FAS	Fas	Anti-apoptosis, immune response, signal transduction
FASLG	Fas ligand	Apoptosis
FGF2	Fibroblast growth factor 2 (basic)	Ras protein signal transduction, angiogenesis, cell differentiation and proliferation
HGF	Hepatocyte growth factor	Growth factor activity
IGF1	Insulin-like growth factor	DNA replication, Ras protein signal transduction
IGFBP1	Insulin-like growth factor binding protein 1	Cell growth, signal transduction
IGFBP3	Insulin-like growth factor binding protein 3	Apoptosis
IGFBP4	Insulin-like growth factor binding protein 4	DNA metabolism, cell proliferation
IGFBP6	Insulin-like growth factor binding protein 6	Regulation of cell growth
ICAM1	Intercellular adhesion molecule 1	Cell-cell adhesion
IFNG	Interferon, gamma	Cell motility, cell growth, immune response
IL1RN	Interleukin 1 receptor antagonist	Inflammatory response
IL1R1	Interleukin 1 receptor, type I	Signal transduction, inflammatory response
IL1R2	Interleukin 1 receptor, type II	Immune response
IL1B	Interleukin 1, beta	Antimicrobial humoral response, apoptosis, cell proliferation
IL10	Interleukin 10	B cell differentiation and proliferation
IL12A	Interluekin 12A	T-helper cell differentiation, cytokine activity, antimicrobial humoral response
IL12B	Interleukin 12B	T-helper cell differentiation, cytokine activity, antimicrobial humoral response
IL13	Interleukin 13	Antimicrobial humoral response, cell motility and proliferation
IL17A	Interleukin 17A	Apoptosis, cell-cell signaling, cytokine activity
IL18	Interleukin 18	T-helper 1 type immune response, angiogenesis
IL2RA	Interleukin 2 receptor, alpha	Apoptosis, cell proliferation
IL4	Interleukin 4	B cell differentiation, T-helper 2 type immune response
IL5	Interleukin 5	Cytokine acitivity, inflammatory response
IL6	Interleukin 6	B cell differentiation, acute-phase response
IL6R	Interleukin 6 receptor	Cell proliferation
IL8	Interleukin 8	G-protein coupled receptor protein signaling pathway
LEP	Leptin	Cell-cell signaling
LTA	Lymphotoxin alpha	Cell-cell signaling, apoptosis, immune response
MIF	Macrophage migration inhibitory factor	Cell proliferation, inflammatory response, anti-apoptosis
MMP1/TIMP1 complex	Matrix metallopeptidase 1/Tissue inhibitor of metallopeptidase 1 complex	Regulation of collagen catabolism
MMP1/TIMP2 complex	Matrix metallopeptidase 2/Tissue inhibitor of metallopeptidase 2 complex	Regulation of collagen catabolism
MMP10	Matrix metallopeptidase 10	Collagen catabolism
MMP2	Matrix metallopeptidase 2	Calcium ion binding, collagen catabolism
MMP9	Matrix metallopeptidase 9	Collagen catabolism
PAPPA	Pregnancy-associated plasma protein A, pappalysin 1	Cell differentiation
PDGF	Platelet derived growth factor	Cell proliferation, cell-cell signaling, regulation of cell cycle
RETN	Resistin	Unknown
SELE	Selectin E	Cell adhesion, inflammatory response
SELL	Selectin L	Cell adhesion and motility
TIMP1	TIMP metallopeptidase inhibitor 1	Enzyme inhibitor activity
TIMP2	TIMP metallopeptidase inhibitor 2	Enzyme inhibitor activity
TNF	Tumor necrosis factor	Regulates cell differentiation, proliferation and apoptosis, cell-cell signaling, inflammatory response
TNFRSF11B	Tumor necrosis factor receptor superfamily, member 11b	Apoptosis, cytokine activity
TNFRSF1A	Tumor necrosis factor receptor superfamily, member 1A	Apoptosis, enzyme binding
VCAM1	Vascular cell adhesion molecule 1	Cell-cell adhesion inflammatory response
VEGF	Vascular endothelial growth factor	Angiogenesis

**Table 3:** Antigen titration data. Data for a subset of the antibody probes on the array is shown. Sensitivity was tested by applying dilutions of pure antigens in PBS buffer and measuring the resultant fluorescence signal (see Supplemental Data for sample titration curves). Bold indicate the approximate limit of detection for that protein, meaning that lower antigen levels were not detected. Other numbers are nominal limits reflecting the lowest concentration tested; the actual limit of detection for these proteins may be lower.

Probe name	L.O.D. (pg/ml)
ADIPOQ	25
CCL1	200
CCL13	1400
CCL18	5000
CCL22	280
CCL28	240
CCL5	170
CCL7	20
CCL8	400
CSF1	400
CXCL10	160
CXCL11	25
CXCL9	5000
FAS	69
FASLG	22
IGFBP1	19
IGFBP4	4000
IL1B	3
IL1RN	1400
IL1R2	188
IL1R1	100
IL10	840
IL12A	268
IL18	75
IL2RA	39
IL4	100
IL5	11
IL6	200
IL6R	36
IL8	266
LTA	200
MIF	240
MMP10	200
MMP9	650
RETN	19
SELE	750
TNF	4100
TNFRSF11B	71
VCAM1	10

**Table 4:** Top scoring proteins that significantly differentiate HCC from hepatitis.

Probe	t-test score p value	Ratio fold change (HCC/hepatitis)
1. AFP	0.003	2.40
2. CTNNB	0.031	3.51
3. CSF1	0.036	1.46
4. SELL	0.002	−1.21
5. IGFBP6	0.014	−1.15
6. IL6R	0.020	−1.10
7. VCAM1	0.046	−1.07

**Table 5:** Top scoring proteins that significantly differentiate HCC with AFP < 20 ng/ml from hepatitis.

Probe	t-test score p value	Ratio fold change (HCC/hepatitis)
1. IL1RN	0.006	1.96
2. IFNG	0.009	1.80
3. CDKN1A	0.019	1.67
4. RETN	0.019	1.85
5. CXCL14	0.022	1.65
6. CTNNB	0.026	3.91
7. FGF2	0.021	−3.61
8. SELL	0.039	−1.19
